# Effect of Cellulose Nanocrystals and Lignin Nanoparticles on Mechanical, Antioxidant and Water Vapour Barrier Properties of Glutaraldehyde Crosslinked PVA Films

**DOI:** 10.3390/polym12061364

**Published:** 2020-06-17

**Authors:** Weijun Yang, Guochuang Qi, José Maria Kenny, Debora Puglia, Piming Ma

**Affiliations:** 1 The Key Laboratory of Synthetic and Biological Colloids, Ministry of Education, Jiangnan University, Wuxi 214122, China; weijun.yang@jiangnan.edu.cn (W.Y.); guochuang_qi@163.com (G.Q.); p.ma@jiangnan.edu.cn (P.M.); 2 Materials Engineering Center, Civil and Environmental Engineering Department, University of Perugia, UdR INSTM, 05100 Terni, Italy

**Keywords:** polyvinyl (alcohol), glutaraldehyde, cellulose nanocrystals, lignin nanoparticles

## Abstract

In this work, PVA nanocomposite films containing cellulose nanocrystals (CNC) and different amounts of lignin nanoparticles (LNP), prepared via a facile solvent cast method, were crosslinked by adding glutaraldehyde (GD). The primary objective was to investigate the effects of crosslinker and bio-based nanofillers loading on thermal, mechanical, antioxidant and water barrier behaviour of PVA nanocomposite films for active food packaging. Thermogravimetric analysis showed improved thermal stability, due to the strong interactions between LNP, CNC and PVA in the presence of GD, while Wide-angle X-ray diffraction results confirmed a negative effect on crystallinity, due to enhanced crosslinking interactions between the nanofillers and PVA matrix. Meanwhile, the tensile strength of PVA-2CNC-1LNP increased from 26 for neat PVA to 35.4 MPa, without sacrificing the ductility, which could be explained by a sacrificial hydrogen bond reinforcing mechanism induced by spherical-like LNP. UV irradiation shielding effect was detected for LNP containing PVA films, also migrating ingredients from PVA nanocomposite films induced radical scavenging activity (RSA) in the produced films in presence of LNP. Furthermore, PVA-CNC-LNP films crosslinked by GD showed marked barrier ability to water vapour.

## 1. Introduction

Lignocellulosic materials, as the most abundant biopolymers and naturally available renewable resources, easily found in territorial plants, have been widely investigated. They are a balanced mixture of lignin, cellulose, hemicellulose and tannins, containing specific functionalities that permit their conversion, as alternative materials to fossil fuels, to biofuels and other organic compounds [1,2]. Lignocellulosic materials have found many applications as reactive filler in various polymers, including thermoplastics, such as polyethylene [3,4], poly (lactic acid) [5,6,7], poly (methyl methacrylate) [8,9], polyurethane [10,11,12] etc.) and thermosetting matrices, such as phenolic [13,14] and epoxy resins [15,16,17].

Polyvinyl alcohol (PVA), as a hydrophilic and semi-crystalline polymer, has been widely applied in the food packaging, construction and household sectors, owing to its high performance, including organic excellent biocompatibility and solvent resistance [18,19]. Nevertheless, poor thermal stability, biological activity and high moisture absorption of PVA limit its application as active packaging. To improve its properties and enrich its functionalities, other polymers or biomass reinforcements, such as nanocellulose [20], starch [21,22], chitin/chitosan [23], have been added to PVA.

Limiting the analysis to chemically modified filler and/or crosslinked matrix, examples of anhydride-modified nanocellulosic reinforcements in PVA can be found in the work of Spagnol et al. [24], where cellulose nanowhiskers (CWs) were treated with maleic, succinic, acetic or phthalic anhydride to compare the interaction of the carboxylic groups with PVA films. The addition of 9 wt.% of modified CWs increased the storage modulus up to 4.4 times, compared to the bare PVA films. Oksman et al. [25] also prepared PVA/cellulose nanocrystals (CNC) films crosslinked by sodium tetraborate decahydrate (borax). The results showed that the presence of CNC affected the crystallization behaviour of the crosslinked PVA; meanwhile, the crystallization temperature of the crosslinked PVA with CNC increased considerably from ∼152 to ∼187 °C, consequently improving the thermal stability of the reference system. Sirviö et al. [26] synthesized bifunctional reactive cellulose nanocrystals (RCNC) with carboxyl and aldehyde functionalities, which were used as reinforcements to prepare acetal-bonding cross-linked PVA films. Mechanical, thermal, and barrier properties of PVA-RCNC films with a variable mass ratio of RCNC (0.5–10 wt.%) were evaluated. Tensile strength and Young’s modulus were up to 2-fold greater than those of pure PVA film with the addition of 10 wt.% acetal-bonding RCNC. An addition of only 0.5 wt.%. RCNC improved the tensile strength of the PVA film by 66% and the modulus by 61%, while a significantly lower reinforcing effect (19% with CNC loading of 0.5%) was found using unmodified CNC. Meanwhile, the oxygen barrier and thermal properties PVA were effectively preserved when RCNC were introduced into the films.

Lignin was also incorporated into PVA, inducing overall improvement of mechanical strength and thermal stability, UV irradiation resistance, antioxidant and antimicrobial properties [27,28]. Inspired by natural spider silk, Zhang et al. [29] prepared nanostructured biomimetic polymeric film by directly adding lignosulfonic acid, without any modification, into PVA matrix, reaching high values for toughness (172 J g^−1^) and maintaining strain at break values higher than 280%. The authors believed that this behaviour could be ascribed to the stress-induced scattering of lignin nanoparticles (LNP) and strong intermolecular sacrificial hydrogen bonding interactions. Fang et al. [30] prepared alkali lignin/PVA films cross-linked by using glutaraldehyde as crosslinker and glycerin as plasticizer. Results showed that the realized films had satisfactory mechanical and thermal stability under the optimal reaction conditions, in particular cast films showed improved and adequate mechanical properties with specific mass ratio of PVA, alkali lignin, glutaraldehyde and glycerol. In our previous work [31], we also found that lignin nanoparticles (LNP) and chitosan synergistically worked on the enhancement of antioxidant and antimicrobial response of physically crosslinked PVA films, potentially enriching their applications in packaging and biomedical applications.

Examples of combined use of cellulose and lignin for enhanced antioxidant and mechanical behaviour can be found in Guo et al. [32], where the authors prepared cellulose/technical lignin composite films having UV blocking and antioxidant activities, due to the special chemical structure of lignin, i.e., phenolic hydroxyl groups. In the meantime, they observed that composite films could effectively block the ultraviolet lights especially for UVB region (280–320 nm) at only 5 wt.% lignin content. Sadeghifar [33] prepared cellulose-lignin films by regeneration in acetone, in which the lignin were dispersed homogeneously in the presence of covalently bond between cellulose and lignin. Cellulose film containing 2% lignin showed 100% protection of UV-B and more than 90% of UV-A (320–400 nm). The UV protection of prepared films was persistent when exposed to thermal treatment at 120 °C and UV irradiation. Motivated by these previous works and confident that analogous synergism between lignin and cellulose could be found when incorporated in a polymeric matrix, we prepared here bio-based PVA nanocomposite films, containing different amounts of cellulose nanocrystals and lignin nanoparticles, by adding the glutaraldehyde as crosslinker, via a facile solvent cast method. The aim of this work was to investigate the effects of glutaraldehyde and nanofillers loading, as eco-benign reinforcements, on thermomechanical behaviour, wettability, antioxidant and water vapour transmission properties of PVA nanocomposite films. In detail, the present work aims to elucidate the effect of adding nanocellulosic reinforcements on crosslinking ability of aldehyde treated PVA, with specific reference to properties functional for active use in food packaging sector.

## 2. Materials and Methods

### 2.1. Materials

**Figure 1:**
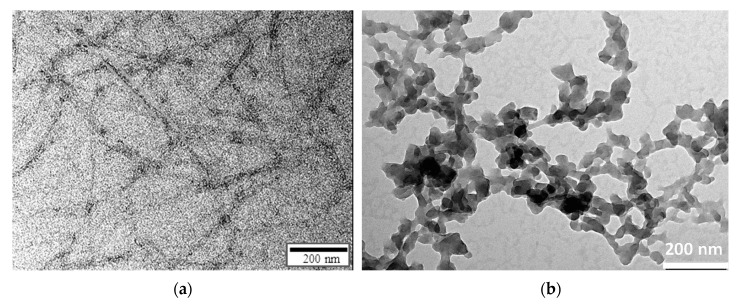
TEM images of CNC (**a**) and LNP (**b**) nanoparticles.

Polyvinyl alcohol (PVA) (M_w_, 120–150 kg mol^−1^) was supplied by Sigma-Aldrich. Microcrystalline cellulose was purchased from Sinopharm Chemical Reagent Co., Ltd., Huangpu, Shanghai, China, cellulose nanocrystals were prepared according to the procedure reported in our previous work [34]. Pristine lignin was kindly provided by Shandong Longlive Bio-technology Co., Ltd. Dezhou, China. The lignin was grinded and then sieved through a 140 mesh, lignin nanoparticles were obtained by acid hydrolysis according to the procedure explained in our previous work [35]. TEM images of CNC and LNP are reported in Figure 1. Glutaraldehyde (GD) and all the other required chemicals were purchased from Sinopharm Chemical Reagent Co., Ltd., Huangpu, Shanghai, China.

### 2.2. Preparation of PVA Nanocomposite Films

**Scheme 1:**
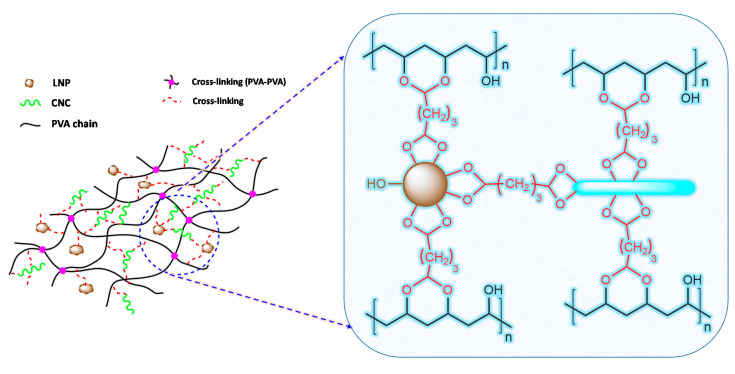
PVA intermolecular cross-linking reaction between PVA chain and CNC, LNP via glutaraldehyde.

Bio-based PVA composite films were prepared by diluting 2.0 g PVA in Millipore water at 2.0% (wt./wt.) and magnetic agitation at 95 °C. After cooling down to 60 °C, PVA solutions were mixed with aqueous suspensions containing various contents of CNC and LNP and sonicated for 5 min. ~0.6 mmol of glutaraldehyde was used as crosslinker and added into well mixed dispersions for further stirring (2 min). Lastly, the dispersions were cast in Petri dishes and dried in the oven at 50 °C for 12 h. The obtained films were labelled as PVA, PVA-GD, P-xCNC-yLNP, where GD represents glutaraldehyde, while x and y mean the weight percentage of the reinforcements in PVA. The films were peeled off and stored at 53% relative humidity and 25 °C, prior to all characterizations. Intermolecular possible reactions between PVA, CNC and LNP have been schematically shown in Scheme 1.

### 2.3. PVA Nanocomposite Film Characterization

#### 2.3.1. Fourier-Transform Infrared (FT-IR) Spectroscopy

Intermolecular interactions between PVA, LNP and CNC crosslinked in presence of GD were recorded by Fourier transform infrared spectroscopy (Nicolet 6700, Thermo Scientific, Waltham, MA, USA) via attenuated total reflectance accessory (ATR) mode in the range 4000–600 cm^−1^, with a resolution of 4 cm^−1^.

#### 2.3.2. Thermogravimetric Analysis (TGA)

TGA tests were performed by using a thermo gravimetric analyser (TGA, Q500 TA instrument, New Castle, PA, USA). Samples (5 mg) were dynamically heated from 30 to 600 °C under nitrogen atmosphere at 10 °C/min. Values of residual mass at 600 °C were measured in TG curves, while temperatures for 1st peak (T_p1_) and maximum degradation rate (T_d_) were collected from derivative curves (DTG).

#### 2.3.3. Wide-Angle X-Ray Diffraction (WAXD)

Patterns of samples were recorded on an X-ray diffractometer (Bruker AXS D8, Bruker Beijing Scientific Technology Co., Beijing, China) with 2θ = 5°−40° at 40 kV and 40 mA.

#### 2.3.4. Tensile Properties

Tensile tests of the films were carried out by using an Instron 5967 (Instron, Norwood, MA, USA) tensile tester, in accordance with ISO 527-1 standard (500 N load cell and a 5 mm/min crosshead speed were used). Rectangular samples (1 × 5 cm^2^) were conditioned at room temperature in a box with Mg(NO_3_)_2_ saturated solution (53% RH). After equilibration, the films were tested at RT (at least six duplicates for each formula were considered) and tensile strength and elongation at break were evaluated from resulting curves.

#### 2.3.5. Microstructure

Morphology of cross-sections for PVA nanocomposite films was analysed via using a Hitachi S-4800 Scanning Electron Microscope (Hitachi Technologies Corporation, Tokyo, Japan). Film specimens were cryo-fractured in liquid nitrogen and the surfaces were then gold coated and observed by using an accelerating voltage of 5 kV.

#### 2.3.6. Optical Properties

Light transmittance of PVA nanocomposite films (normalized thickness of 80 µm) was measured by using a UV-Vis spectrometer (SHIMADZU UV-3600 plus, Shimadzu Co. Ltd., Beijing, China), performing a scan from 250 to 900 nm at 240 nm/min.

#### 2.3.7. Wettability

Wettability of PVA nanocomposite films was measured by using a contact meter (FTA 1000, First Ten Angstroms, Inc., Portsmouth, NH, USA) instrument coupled with a Drop Shape Analysis software. Water contact angle (WCA) measurements (average values of three) were done at room temperature under static conditions and values were recorded for 25 s at 3 s intervals.

#### 2.3.8. Overall Migration Activity

Overall migration measurements were carried out in simulant D3 (isooctane). Rectangular samples of 10 cm^2^ were maintained in the isooctane solution (10 mL) in standard conditions for 2 days at 20 °C. Value of migrated substances (mg/kg) of the simulant was determined by weighing the residues with an analytical balance (±0.01 mg precision).

#### 2.3.9. Antioxidant Activity

DPPH radical scavenging activity for PVA composites films was checked in accordance with modified methods from Byun et al. [36] and Domenek et al. [37]. Films (0.2 g) cut into small pieces were immersed in 5 mL of ethanol for 24 h at room temperature, after that collected supernatant was tested for evaluation of DPPH radical scavenging activity. In details, methanol extract (3 mL) was mixed with DPPH in methanol (3 mL, 50 mg/L) obtaining a final concentration solution of 25 mg/L for DPPH. The mixtures were maintained at RT in darkness for 1 h; after that, absorbance values were measured at 517 nm using a UV-Vis spectrometer (SHIMADZU UV-3600 plus, Shimadzu Co. Ltd., Beijing, China), by setting as control the DPPH methanol solution extracted from neat PVA. DPPH radical scavenging activity was calculated by Equation (1), where A_sample_ was the absorbance of sample and A_control_ was the absorbance of the control.

RSA (%) = (A_control_ − A_sample_)/A_control_ × 100 (1)

#### 2.3.10. Water Vapour Transmission Rate (WVTR)

Water vapour transmission rate of the films was measured using a water vapour transmittance tester (PERME W3/060, Labthink Instruments Co., Ltd., Jinan, China) at 38 °C and 98% relative humidity (RH) on duplicate samples with a diameter of 10.0 cm for each formulation.

## 3. Results

### 3.1. FT-IR Analysis

**Figure 2:**
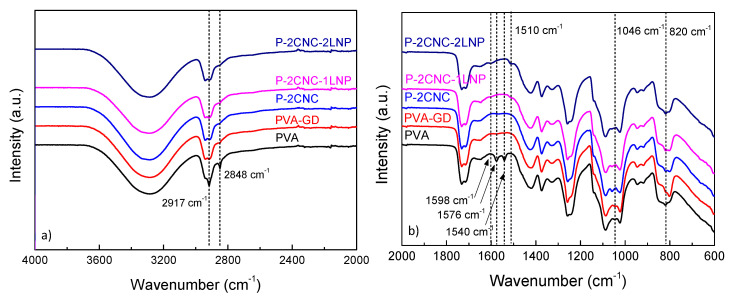
FTIR spectra of neat PVA (Ctrl), PVA-GD, P-2CNC, P-2CNC-1LNP and P-2CNC-2LNP.

The intermolecular interactions between neat PVA and crosslinker in presence of CNC and LNP in nanocomposite films were studied by FT-IR spectroscopy, related spectra are reported in Figure 2. The neat PVA spectrum exhibits characteristic peak at 1645 cm^−1^, associated with stretching and deformation vibration of OH groups, potentially sensitive to intermolecular interactions [31]. Peaks at 1329, 1086 and 1043 cm^−1^ can be associated with the deformation vibration of C–O groups [38]. It is expected that crosslinking of PVA by GD decreases the number of hydroxyl groups by forming crosslink ester bonds in the PVA, with variations in intensity for −OH and −C = O (ester) group [39]. Disappearance of peaks at 1576 and 1540 cm^−1^, due to deformation of CH bonds, can be an indication of possible crosslinking reaction.

The presence of cellulose nanocrystals in PVA matrix also leads to a variation in the intensity of OH stretching, attributable to the interaction between OH group on the surface of CNC and the group OH in the PVA matrix. The addition of CNC also resulted in the appearance of a weak signal at 1163 cm^−1^, which corresponds to the asymmetric ring breathing mode of cellulose, and it is still present in the samples containing both CNC and LNP. A new band at 1060 cm^−1^, corresponding to the COH bending vibrations of alcohol groups present in cellulose, was also detected [40]. The peak at 1043 cm^−1^ can be assigned to C-O-C pyranose ring vibration, which is superposed with the C–O ether bond of the PVA polymer chain. The peak at 1730 cm^−1^ is due to the stretching of carbonyl group in PVA.

When LNP were added to the PVA-2CNC system, new signals at 1598 and 1510 cm^−1^ appeared, due to the C = C stretching of aromatic rings of lignin, in the meanwhile intramolecular interaction of GD with LNP functional groups was detected and supported by the modification of peaks associated with the stretching vibrations of -C-O-C and −CH of aldehyde group at 2917 and 2848 cm^−1^, forming acetal groups [41,42]. The peak at 3392 cm^−1^, due to stretching vibration of OH groups, was more visible in the PVA-2CNC-2LNP sample, supporting the existence of intermolecular reaction of GD and PVA [43]. Differences can be found in the intensities of peaks at 1258 cm^−1^ (C–O bending) and 1241 cm^−1^ (C-O stretching), while the signal at 802 and 820 cm^−1^, associated with CH out of the plane deformation, shifted to higher wavenumbers for all the samples containing lignin in the presence of GD.

### 3.2. Thermogravimetric Analysis (TGA)

**Figure 3:**
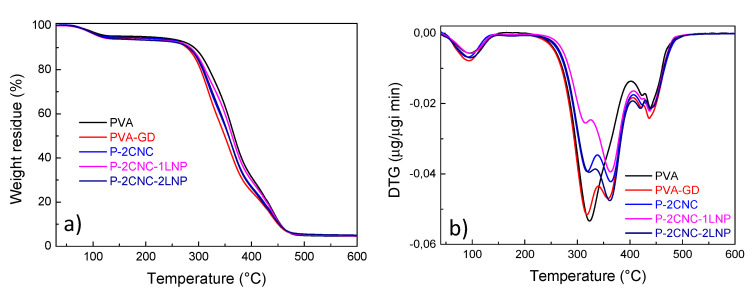
TG (**a**) and DTG curves (**b**) of PVA, PVA-GD, P-2CNC, P-2CNC-1LNP and P-2CNC-2LNP films.

TG and DTG curves of PVA and PVA nanocomposite films, reported in Figure 3, show the effect of CNC and LNP loading on the thermal stability of GD crosslinked PVA films. Thermal parameters including T_p1_, T_d_ and residual mass determined at 600 °C are collected in Table 1. As already shown by Sonker et al. [39], degradation of neat PVA can be found in three temperature ranges, 50~150, 275~400 and 425~500 °C. All the materials start to lose weight at about 50 °C, due to the evaporation of moisture. As evidenced in Figure 3 and Table 1, the moisture release was modified in the case of crosslinked films, with a shift towards higher temperatures of T_p1_ peak (T_p1_ was determined from DTG curves as the temperature when moisture evaporation reached the maximum). The second and the third degradation temperatures correspond to the degradation of neat PVA; in particular, the second/main degradation step of PVA film corresponds to the chain scissoring, with the removal of residual acetate groups, due to the incomplete hydrolysis of PVA that remains in the chains, while the third degradation step is due to the cyclization reaction and continual elimination of residual acetate groups [44]. The second peak (T_d1_) and its shoulder (T_d2_) slightly shifted to a lower temperature for the crosslinked membranes in comparison with the pure PVA film; additionally, the crosslinking reaction with GD better evidenced the shoulder peak at 360 °C. With addition of CNC and LNP nanofillers, the decomposition curves were somewhat modified; in particular, T_d2_ increased from 356 °C (PVA-GD) to 364 °C (P-2CNC) and 365 °C (P-2CNC-1LNP), demonstrating an enhanced thermal stability due to a more stable crosslinked network, especially when LNP was added to the system.

This could be interpreted by considering that CNC and LNP are able to enhance cross-linking interactions, so motion of the PVA chains within the interfaces was subsequently depressed [45]. The evidence of retained weight loss (increased weight loss from 4.6 to 5.1%) also confirmed the overall enhanced thermal stability of the GD crosslinked PVA systems in presence of thermally stable lignocellulosic nanofillers.

### 3.3. WAXD

**Figure 4:**
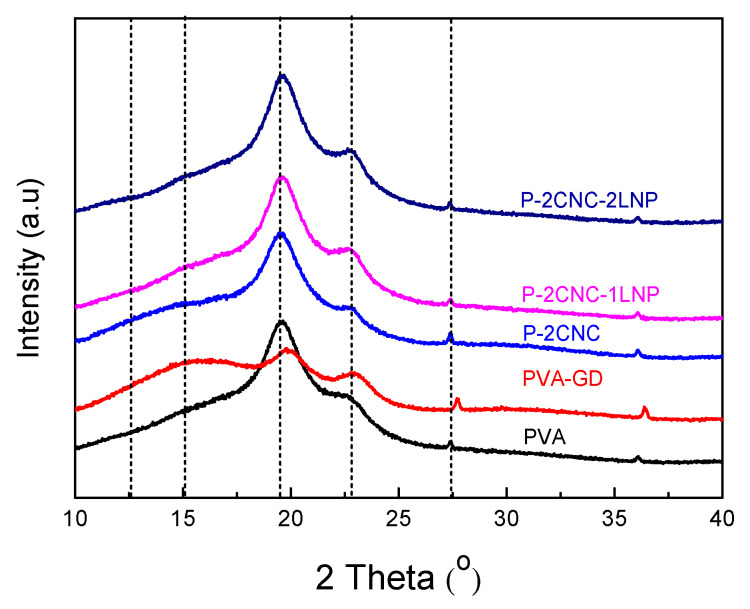
WAXD curves for neat PVA and PVA nanocomposites.

The results of WAXD analysis of neat PVA and PVA nanocomposites films are reported in Figure 4. The two main diffraction peaks at 2θ = 19.8° and 22.8° for pure PVA are associated with the (101) and (200) orthorhombic lattice structure planes, respectively. Meanwhile, the peak at 2θ = 19.8° was responsible of presences for strong inter- and intra-molecular H bonding [46,47]. It is noted that the diffraction peaks of PVA-GD declined, suggesting that the well-organized PVA chain in the crystalline section could be destructed, due to the presence of GD. However, the characteristic diffraction peaks of PVA also appear in the diffraction patterns of all the PVA nanocomposites, suggesting that the crystal structure of PVA was essentially recovered with the introduction of CNC and LNP. In the case of P-2CNC, in correspondence of main diffraction peaks for CNC (2θ = 14.8°, 16.6° and 22.9°), the PVA signal showed a different shape. When lignin, having a three-dimensional amorphous structure [29], was added to the film, no variation was found in the spectrum. In the cases of CNC/LNP-containing systems, the diffraction peak at 2θ = 19.8° did not change significantly, while the peak intensity at 2θ = 22.8° slightly and gradually increased in comparison with pure PVA, indicating that the crystalline region was enhanced. Evaluation of crystallinity degree values from partial areas confirms that crosslinking reduced the crystallinity (from 39.9% for neat PVA to 25.4% for PVA-GD), while the introduction of CNC and LNP partially recovered it (mean value of 30.9% for P-2CNC, 30.5% for P-2CNC-1LNP and 31.2% for P-2CNC-2LNP).

### 3.4. Tensile Properties

**Figure 5:**
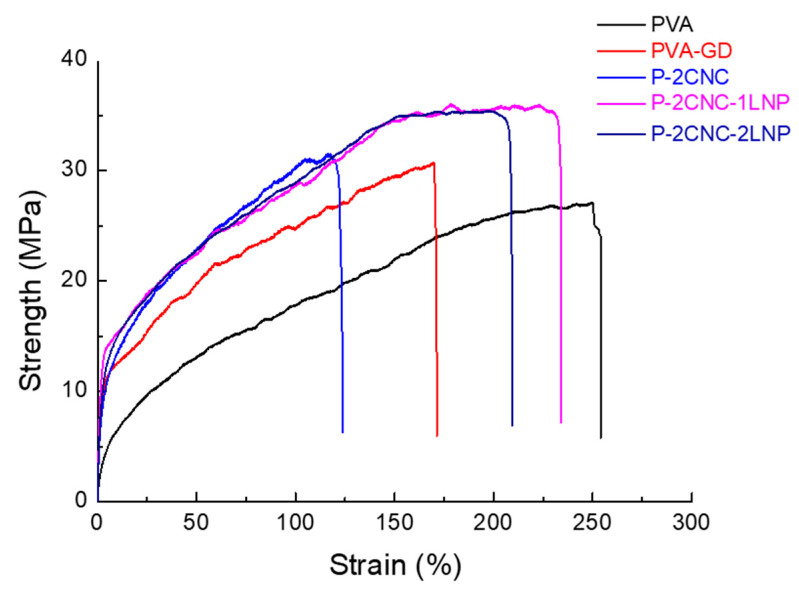
Stress–strain curves of PVA and PVA nanocomposite films.

The typical stress–strain curves obtained from tensile measurements of PVA nanocomposite films are reported in Figure 5, while values for tensile strength and elongation at break are included in Table 2.

**Scheme 2:**
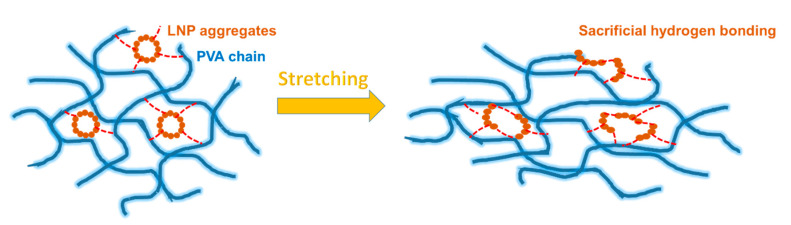
Schematic sacrificial hydrogen bond reinforcing mechanism of LNP in PVA film.

As reported in previous studies [26,31], when PVA systems are loaded with CNC and LNP, a growth in tensile strength and decrease in elongation at break can be observed when compared with neat PVA films. The addition of CNC and cross-linking reaction with GD restricted the polymer motion, with a consequent increase in tensile strength and decrease in elongation at break in PVA-GD and P-2CNC films [48]. When LNP are added to PVA-2CNC, they may promote the formation of cross-links. Interestingly, strength and ductility were simultaneously enhanced, as the tensile strength and elongation at break reached 35.4 MPa and 234%, respectively. This could be explained by the reinforcing mechanism of strain-induced scattering and three-dimensional enmeshment effect of LNP on PVA chain segments, as shown in Scheme 2. When the PVA nanocomposite film is stretched, the secondary spherical particles are destroyed and the LNP primary aggregates gradually scatter in the PVA matrix, so confined intermolecular H-bonds dynamically fracture and reform. Meanwhile, the amorphous PVA chain segments are constrained by the 3D LNP molecules via H-bonds as the anchor points, which promote the chain extension and alignment upon stretching in PVA-CNC-LNP systems. Through the strain-induced scattering and 3D enmeshment effect of LNP on PVA segments via strong intermolecular sacrificial H-bonds, the external load energy can be efficiently dissipated, the stress concentration is then retarded, and the initiation of micro cracks is postponed, thus eventually reinforcing the strength, toughness, and ductility of the composite film [29].

### 3.5. Microstructure

**Figure 6:**
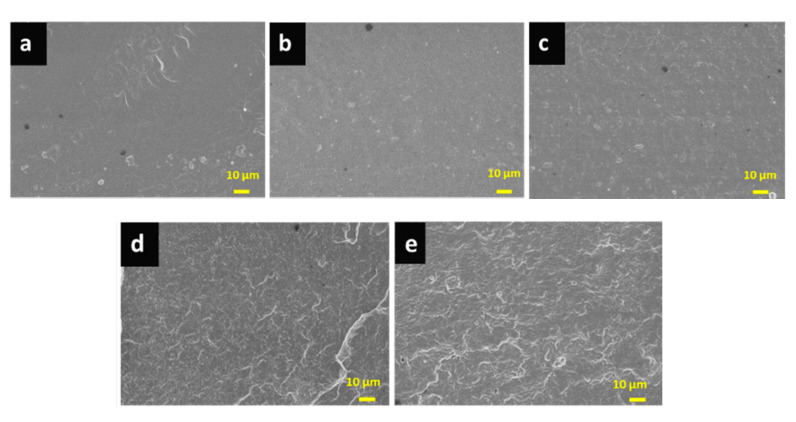
Fractured surfaces morphologies for representative PVA (**a**), PVA-GD (**b**), P-2CNC (**c**), P-2CNC-1LNP (**d**) and P-2CNC-2LNP (**e**).

The morphology of the cross-section of the PVA films at different weight amounts of CNC and LNP was analysed via FESEM; micrographs are also included in Figure 6.

It can be seen that a relatively smooth fractured surface can be observed in the pure PVA and PVA-GD films. CNC incorporation reduced the ductility of the material, as is clearly evident in the corresponding surface microstructure, essentially ascribed to the strong crosslinking and hydrogen bonding interactions. However, in the case of P-2CNC-1LNP and P-2CNC-2LNP, some plastic deformations of PVA matrix can be observed, implying an improved ductility. This result can be interpreted on the basis of the improved compatibility between LNP and PVA, due to interfacial reactions between the hydroxyl groups of both LNP and PVA matrix. Meanwhile, adding LNP into a PVA system may form strong and tough film via nanostructured microphase separation and intermolecular sacrificial hydrogen bond interactions. [29] As a result, enhancement in toughness and tensile strength for PVA film could be observed. No agglomeration or macroscopic phase separation were observed, demonstrating good dispersion of CNC and LNP in PVA matrix.

### 3.6. Wettability

**Figure 7:**
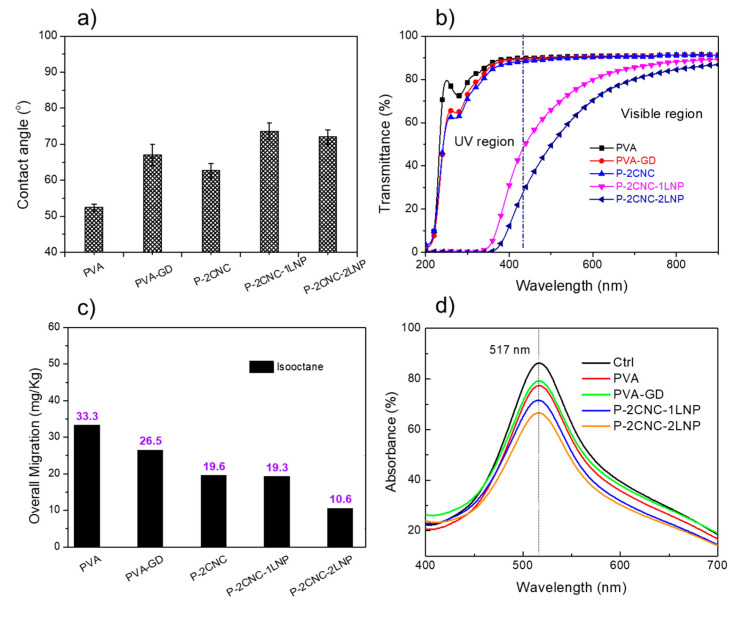
(**a**) Water contact angle of different PVA composite films; (**b**) UV transmittance of PVA nanocomposite films; (**c**) Overall migration values of different PVA nanocomposite films in isooctane solution and (**d**) Antioxidant property of migrated ingredients from different PVA nanocomposite films.

Figure 7a showed the water contact angle (WCA) for the PVA nanocomposite films crosslinked by glutaraldehyde. Overall, PVA shows a rather high hydrophilicity surface (WCA = 52.4°), due to presence of abundant hydroxyl groups on surface. On the contrary, because of the consumption of the hydroxyl groups during the crosslinking, the PVA-GD film showed higher WCA (67°) in the presence of glutaraldehyde, which was partially modified again by adding CNC, bringing OH functionality, to the film (62.7°). Meanwhile, WCA values increased gradually again with increasing amount of LNP nanofiller, probably due to the intrinsic hydrophobicity of alkali lignin [49]. This result illustrates that the PVA nanocomposites films crosslinked by glutaraldehyde will reduce the water accessibility of PVA, possibly extending its application for aqueous and alcoholic-based foods contactable packaging films.

### 3.7. Optical Properties

UV irradiation effects need to be taken in account when polymer films are used in food packaging, especially in some UV-sensitive food, as the UV light would accelerate the spoilage of food. Although PVA has been widespread considered as a packaging material, its unsatisfactory UV shielding behaviour still needs to be enhanced to extend the application. The optical properties of all PVA nanocomposite films with various amounts of CNC and LNP are shown in Figure 7b. CNC has weak UV resistance behaviour, while lignin has exhibited excellent UV resistance behaviour in some studies [6,33]. All PVA nanocomposite films remain highly transparent in visible spectrum regions (above 400 nm). As expected, the optical transparency in UV spectrum regions (below 400 nm) decreased gradually until 0 when only 1 wt.% of LNP was incorporated. It could be also observed that the P-2CNC-2LNP films have more significant UV resistance behaviour.

### 3.8. Overall Migration and Antioxidant Activity of Migrating Substances

Overall migration measurements in food simulants D3 were examined and the results are given in Figure 7c. Neat PVA film showed a high migration value of 33.3 mg kg^−1^. Interestingly, when CNC and LNP were incorporated in crosslinked films, the overall migration values decreased, with a migration level of 10.6 mg kg^−1^ of simulant measured for the P-2CNC-2LNP system. It is proposed that the formed PVA/CNC/LNP crosslinked network will prevent the migration effect of the ingredients, as a consequence of lowering the migration values. It should be noted that these overall migration values are well below the regulated limits (60 mg/kg) for food contactable film simulants, established by the current European legislation EN No. 10/2011, which directs that polymer materials shall not transmit their component/constituents toward foods exceeding the given migration limits, thus avoiding human health issues, especially for infants.

Free radicals originating from O_2_ exist ubiquitously, or can be created by UV irradiation [50]. These radicals serve as initiators of the chain oxidation of lipids, inducing the spoilage of food. It is, therefore, significant to scavenge these radicals from the head source, as an alternative route to protect foods and prolong their shelf life. Two strategies are frequently considered in active packaging, including: (1) the design of active compound releasing systems; and (2) undesigning constituent scavenging systems [51]. In the first strategy, low-molecular weight nontoxic free radical scavenging active substances, generated from the packaging film, migrate into the foodstuff. For the second strategy, the radical scavenging activity occurs upon their contact with the foodstuff, without the release of active compounds [52]. In this work, the first strategy was designed and considered for the realization of active packaging films. Figure 7d and Table 3 show the radical scavenging activity (RSA) of migrating ingredients from PVA nanocomposite films incubated in ethanol solution for 24 h.

A neat PVA sample was considered as control and did not show any RSA effect. PVA-GD and P-2CNC films had weak RSA responses, with a small reduction of absorbance at 517 nm being observed. As expected, a more remarkable reduction of absorbance at 517 nm and obvious radical scavenging activity was detected with increasing LNP content: 17.2% of RSA for P-2CNC-1LNP and 23.1% of RSA for P-2CNC-2LNP were obtained, respectively. PVA nanocomposite films crosslinked by glutaraldehyde containing LNP can indeed be considered active enough to be used as antioxidant food packaging materials.

### 3.9. WVTR

**Figure 8:**
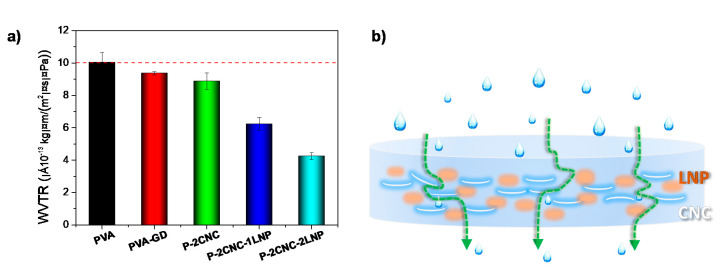
(**a**) WVTR properties of PVA nanocomposite films and (**b**) schematic illustration of the tortuous pathway for water vapour.

Fresh food quality loss is mostly caused by the poor gas barrier properties of polymeric packaging materials. Thus, it is of significance to improve the gas barrier properties of polymeric material films in the packaging industry. The WVTR values of the PVA composites films with different amount of CNC and LNP are shown in Figure 8.

The WVTR value of neat PVA was 1.1 × 10^−13^ kg m/m^2^ s Pa, while the PVA-GD decreased to 0.94 × 10^−13^ kg m/m^2^ s Pa, suggesting intramolecular crosslinking between PVA molecular chains. Clearly, as CNC were added, the WVTR decreased to 0.89 × 10^−13^ kg m/m^2^ s Pa. Furthermore, the WVTR could be reduced to 0.42 × 10^−13^ kg m/m^2^ s Pa when another 2 wt.% of LNP was loaded. This could be interpreted as being on the basis of the modified WVTR pathway. In composite films, the nanofillers serve as impermeable physical barriers in the PLA matrix, and then form a tortuous pathway and force water vapour to swing around them to pass through a more tortuous route, resulting in the improvement of gas barrier performance [53]. On the other hand, the well-dispersed CNC and LNP and excellent synergistic interactions between the nanofillers and PVA in the presence of glutaraldehyde better acted in modifying significantly the tortuous paths, as schematically illustrated in Figure 8b. Consequently, the diffusion paths of water molecules throughout PVA composite films reduced.

## 4. Conclusions

In the present work, PVA nanocomposite films containing different amounts of cellulose nanocrystals (CNC) and lignin nanoparticles (LNP), prepared via a facile solvent cast method and crosslinked by adding glutaraldehyde (GD), were produced. The results showed that nanofillers and crosslinker modified the thermal stability, mechanical and wettability behaviour of the PVA nanocomposite films, especially in the case of LNP-containing films, due to improved crosslinking interactions between PVA and LNP. The crosslinked PVA-CNC-LNP films showed improved tensile strength without sacrificing the ductility, which could be explained by the reinforcing mechanism of strain-induced scattering and three-dimensional enmeshment effect of LNP on the PVA chain. The UV shielding performance and the radical scavenging activity (RSA) of migrating ingredients for LNP containing PVA nanocomposites were also enhanced. Additionally, the nanofillers could serve as impermeable physical barriers to form a tortuous pathway and force water vapour to pass through a more tortuous route, resulting in the improvement of water barrier performance. In conclusion, the produced PVA films can be considered as competitive active UV-resistant and antioxidant food packaging products.

## Figures and Tables

**Table 1:** Thermal decomposition temperature of representative PVA and PVA-based nanocomposite films, the residue calculated at 600 °C.

Samples	T_p1_ (°C)	T_d1_ (°C)	T_d2_ (°C)	Residual Mass (%)
PVA	94.0 ± 0.2	323 ± 1.4	-	4.6 ± 0.1
PVA-GD	94.3 ± 0.1	318 ± 1.0	356 ± 1.4	4.8 ± 0.2
P-2CNC	93.8 ± 0.3	317 ± 0.8	364 ± 0.8	4.8 ± 0.1
P-2CNC-1LNP	97.0 ± 0.2	315 ± 0.5	365 ± 0.6	4.7 ± 0.2
P-2CNC-2LNP	97.2 ± 0.3	320 ± 1.2	363 ± 0.6	5.1 ± 0.1

**Table 2:** Results from tensile tests of PVA crosslinked films in presence of CNC and LNP.

Samples	Tensile Strength (MPa)	Elongation at Break (%)
PVA	26.0 ± 3.4	232 ± 50
PVA-GD	29.0 ± 2.2	170 ± 20
P-2CNC	31.6 ± 1.9	121 ± 20
P-2CNC-1LNP	35.4 ± 2.2	234 ± 30
P-2CNC-2LNP	35.2 ± 1.5	207 ± 35

**Table 3:** Radical scavenging activity (RSA) of migrating ingredients of different PVA nanocomposite films.

Samples	Absorbance (%) @517 nm	RSA (%)
PVA	86.5	0
PVA-GD	77.2	10.7
P-2CNC	79.1	8.6
P-2CNC-1LNP	71.6	17.2
P-2CNC-2LNP	66.5	23.1
